# A Comparative Study of the Efficacy of Chemical Peels and Microneedling in the Treatment of Moderate to Severe Melasma

**DOI:** 10.7759/cureus.98962

**Published:** 2025-12-11

**Authors:** Aamna Batool, Ali Jaaen Seger, Nazia Akhtar, Sahar Mateen, Muhammad Usman Amiruddin, Mehak Zain

**Affiliations:** 1 Dermatology/Medicine, Shifa College of Medicine, Shifa Tameer-E-Millat University, Islamabad, PAK; 2 Pharmaceutical Chemistry, Lecturer Islamic University, Najaf, IRQ; 3 Dermatology, Alnafees Medical College and Hospital, Islamabad, PAK; 4 Dermatology, Dow University of Health Sciences, Civil Hospital Karachi, Karachi, PAK; 5 Plastic Surgeon, UA Aesthetics, Lahore, PAK; 6 Medicine, Fatimiyah Hospital, Karachi, PAK

**Keywords:** chemical peel, melasma, microneedling technique, patients satisfaction, treatment

## Abstract

Background: Melasma is a chronic, relapsing hyperpigmentation disorder that commonly affects individuals with darker skin phototypes, leading to significant cosmetic and psychological distress. Despite various treatment options, achieving lasting improvement with minimal side effects remains challenging.

Objective: To compare the efficacy and safety of microneedling with tranexamic acid and vitamin C versus 15% trichloroacetic acid (TCA) chemical peeling in the treatment of moderate to severe melasma.

Methods: This was a comparative interventional study conducted at Shifa International Hospital from November 2024 to June 2025, including 120 patients with moderate to severe melasma, allocated equally into two groups. Group A underwent microneedling with tranexamic acid and vitamin C, while Group B received 15% TCA chemical peels. Modified Melasma Area and Severity Index (MASI) scores, Physician Global Assessment (PGA), and Patient Global Assessment (PtGA) were recorded at baseline, 4, 8, and 12 weeks. Adverse effects were documented. Data were analyzed using appropriate statistical tests, with p<0.05 considered significant.

Results: Both groups showed significant MASI score reductions over 12 weeks (p<0.001 within groups). The microneedling group improved from 9.11 ± 4.09 to 5.21 ± 2.05, while the TCA group improved from 22.97 ± 4.36 to 13.16 ± 4.49. The between-group difference was statistically significant (p = 0.006). By week 12, at least 60% improvement was achieved in 18 patients (30.0%) in the microneedling group compared with 10 patients (16.7%) in the TCA group. Moderate improvement (40-59%) was observed in 22 patients (36.7%) receiving microneedling versus 16 patients (26.7%) receiving TCA peels. Minimal improvement (<20%) was more frequent in the TCA group (14 patients, 23.3%) compared with microneedling (8 patients, 13.3%). Adverse effects were reported by 22 patients (36.7%) in the microneedling group and 35 patients (58.3%) in the TCA group. Temporary redness was most common, affecting 16 patients (26.7%) in the TCA group and 10 patients (16.7%) in the microneedling group. No severe adverse events occurred.

Conclusion: Both 15% TCA chemical peeling and microneedling with tranexamic acid and vitamin C are effective in treating moderate to severe melasma. However, microneedling demonstrated superior pigment reduction, higher patient satisfaction, and fewer side effects. It may therefore be considered a safer and more effective first-line procedural therapy, particularly for individuals with darker skin phototypes prone to postinflammatory hyperpigmentation.

## Introduction

Melasma is a chronic, non-scarring hyperpigmentation disorder of the skin with irregular light-to-dark brown macules and patches that appear on the face and other sun-exposed or non-sun-exposed body parts with irregular pigmentation. Women, especially those of Fitzpatrick skin types III to V, are the more common group affected, and there is a close relationship of the skin condition with hormonal factors, ultraviolet (UV) radiation, heredity, and some drugs [1,2]. As much as melasma is harmless, it presents a serious cosmetic issue that results in psychological problems, social embarrassment, and the loss of quality of life in individuals with melasma [3]. Melasma pathogenesis is gradual and poorly comprehended. It is due to hyperfunctional melanocytes that can create unnecessary melanin and transfer it to keratinocytes, causing pigmentation to appear [4]. The role of UV radiation as an important aggravating factor cannot be overlooked because it promotes melanogenesis and provokes the appearance of oxidative stress, leading to pigmentation aggravation [5]. Hormonal factors, especially estrogen and progesterone, most likely play a role, which explains why there is more prevalence among women, in addition to relating to pregnancy and the use of oral contraceptives [6]. It has also been observed that vascular and dermal inflammatory changes and changes in the basement membrane structure occur in melasma skin; hence, it is believed that there is a complex integration of epidermal and dermal factors [7]. Melasma has a chronic relapsing characteristic, which makes treatment quite difficult. Non-therapeutic agents of conventional first-line therapy include topical depigmenting creams that contain hydroquinone, retinoids, and corticosteroids, usually in combination [8]. Nonetheless, their use is restricted, as it is incomplete and can cause adverse effects, including irritation, and is prone to frequent recurrence [9]. Consequently, chemical peels and microneedling as procedural treatments have become increasingly popular as add-on or alternative therapies [10].

The mechanism of action is that chemical peels trigger regulated exfoliation of the skin due to controlled chemical exfoliation, which encourages regeneration of the epidermis and subsequent dispersion of melanin [11]. There are various peeling agents commonly used in the treatment of melasma, including glycolic acid, salicylic acid, trichloroacetic acid (TCA), and lactic acid. Research has shown them to be effective in pigmentation, especially in epidermal melasma, although not much can be derived from them in dermal or mixed types [12]. There are potential side effects such as postinflammatory hyperpigmentation (PIH), which occurs mostly in darker skin types, and it is essential to select both the agent and the concentration of a peeling agent [13]. Collagen induction therapy, otherwise known as microneedling, refers to the generation of superficial micro-injuries on the skin using fine needles that produce an increase in collagen synthesis, promote dermal remodeling, and enhance the delivery of topically applied agents [14]. Recent studies have indicated that microneedling may be used to increase the deposition of depigmenting agents, refine skin texture, and induce pigment dispersion in melasma [15]. Microneedling, unlike chemical peels, targets the epidermis and dermis and may provide superior outcomes in mixed-type melasma [16]. According to studies, comparative analysis shows that chemical exfoliants or peels and microneedling can potentially lead to a considerable reduction in pigmentation scores, including Modified Melasma Area and Severity Index (MASI), but complications, tolerability issues, and recurrences have been reported in both techniques [17]. The initial effect with chemical peels can be achieved more often than with microneedling, but long-lasting effects can achieve greater results without pigmentary complications [18]. Nonetheless, there are no direct human head-to-head comparisons done in moderate to severe melasma, and well-designed comparative clinical trials are needed to provide evidence-based guidance on treatment choice.

Objective

To compare the efficacy and safety of microneedling with tranexamic acid (TA) and vitamin C versus 15% TCA chemical peeling in the treatment of moderate to severe melasma.

## Materials and methods

This was a comparative interventional study conducted at Shifa International Hospital from November 2024 to June 2025, enrolling 120 patients with moderate to severe melasma by using a non-probability consecutive sampling technique. Patients were allocated into two equal groups of 60 each. Group A received 15% TCA chemical peels, and Group B received microneedling with TA and vitamin C. The sample size was calculated by using the WHO sample-size calculator for comparison of two means, referencing Budamakuntla et al. [19], which compared microneedling with TA to chemical peeling in melasma. Using a 95% confidence level, 80% power, an expected mean difference of two units in MASI-score reduction, and a pooled standard deviation (SD) of four, the required sample size was determined to be 60 participants per group (total = 120). Patients were allocated consecutively according to their enrollment sequence. This was an open-label interventional study in which both patients and treating dermatologists were aware of the assigned treatment due to the visible procedural differences between microneedling and chemical peeling. To minimize assessment bias, a single-blind approach was maintained, and all post-treatment evaluations, including the MASI, were performed by an independent blinded dermatologist who had no knowledge of the treatment allocation. No randomization or allocation concealment was applied, as this was a comparative interventional study conducted within a tertiary outpatient setup. The absence of randomization is acknowledged as a potential source of selection bias; however, equal group sizes and consistent eligibility criteria were maintained to minimize imbalance. All participants were advised to use broad-spectrum sunscreens and moisturizers after the procedure. The study protocol was reviewed and approved by the Institutional Review Board (IRB) of Shifa International Hospital, Islamabad (Approval No. RB/DERM/2024/117).

Inclusion and exclusion criteria

Eligible participants were adults between 18 and 50 years of age with moderate to severe melasma confirmed by Wood's lamp examination. Patients with epidermal or mixed-type melasma were considered suitable for enrollment if they demonstrated willingness to comply with follow-up assessments. Exclusion criteria included pregnancy or lactation, a history of bleeding disorders or use of anticoagulant therapy, hypersensitivity to study medications, and the use of depigmenting treatments within the preceding four weeks. Patients with active skin infections, inflammatory dermatoses, or conditions that could interfere with wound healing, such as keloid tendency or systemic immunosuppression, were also excluded.

Data collection

**Figure 1:**
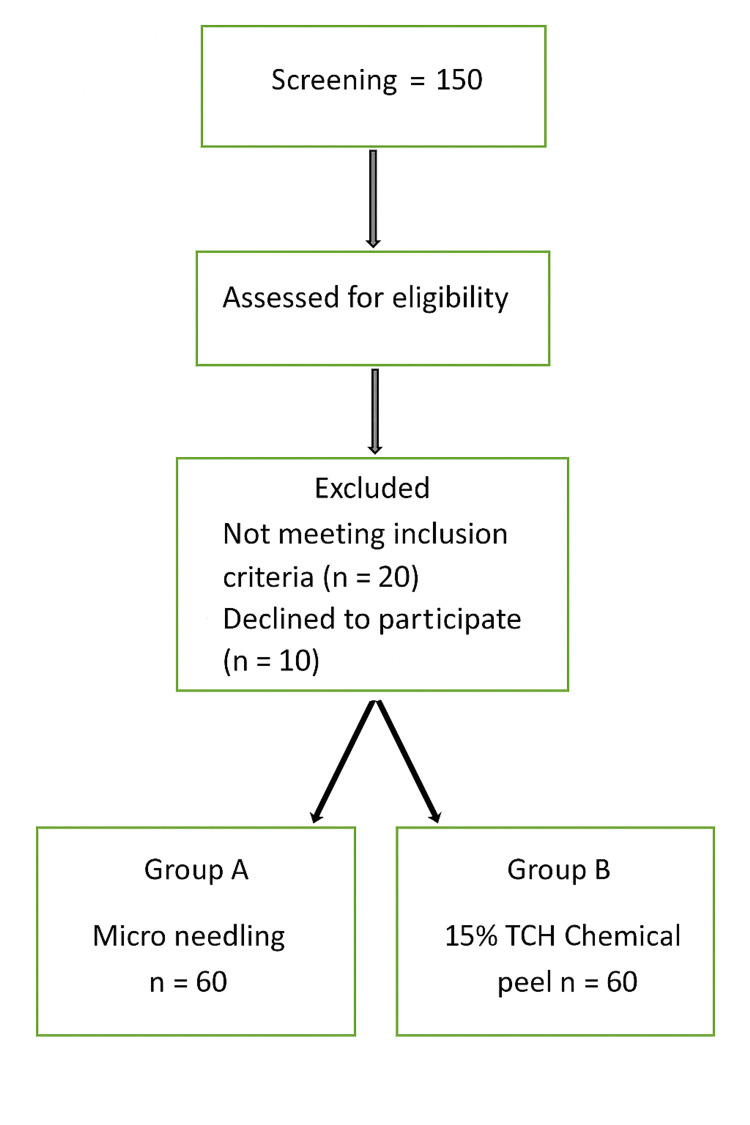
Study flowchart TCH: trichloroacetic acid.

After informed consent was obtained, all participants were evaluated through a detailed clinical history and physical examination, including assessment of melasma type and distribution by using Wood's lamp. Group A participants were treated at a four-weekly frequency for up to three sessions of standardized 15% TCA peels. Under dermatologist supervision, proper neutralization and skin priming by using topical emollients and sunscreen for two weeks before treatment were conducted. Microneedling was performed on Group B patients once every four weeks (three sessions) by using a dermaroller (1.5 mm). Immediately after the procedure, topical application of TA solution (4 mg/mL) and vitamin C (20% solution) was performed to enhance dermal absorption. The post-treatment approach for both groups included advising on sunscreen products and emollient application to reduce any adverse conditions. Data were collected on demographic and clinical characteristics at baseline through a structured interview and clinical examination, including age, sex, and type and distribution pattern. To increase consistency, clinical photographs were collected in a standardized manner at baseline and at the 4-, 8-, and 12-week points by using the same camera and lighting settings. The MASI was used to measure the primary clinical outcome and was scored by a blinded dermatologist. The MASI score, proposed by Kimbrough-Green et al. in 1994, was devised on the pattern of the Psoriasis Area and Severity Index (PASI) [20]. Physician and Patient Global Assessment (PGA, PtGA) [21] were recorded at each follow-up. Any side effects, including erythema, burning, swelling, or itching, were thoroughly documented. A data flow chart is presented in Figure 1.

Statistical analysis

All data were analyzed by using IBM SPSS Statistics for Windows, Version 26 (Released 2018; IBM Corp., Armonk, New York). Continuous variables, including MASI scores, were expressed as mean ± standard deviation (SD). Categorical variables, such as improvement categories and adverse event frequencies, were presented as frequencies and percentages. Within-group comparisons of MASI scores at baseline and subsequent visits were conducted by using the paired t-test. Data normality was assessed by using the Shapiro-Wilk test before applying parametric statistical analyses. A p-value of less than 0.05 was considered statistically significant.

## Results

Data were collected from 120 patients. The mean age in the microneedling group was 38.9 ± 7.1 years, while the chemical peel group averaged 39.6 ± 6.8 years. The majority of participants were middle-aged adults, with the largest subgroup aged 41-50 years (38.3%), followed by 31-40 years (35.0%), while younger adults (18-30 years) accounted for 15.0%. The overall mean age was 39.3 ± 7.0 years. Females formed a predominant portion of the cohort (88.3%), reflecting the higher prevalence of melasma among women. In terms of clinical presentation, the centrofacial pattern was the most frequent type, seen in 65.0% of participants, followed by the malar pattern in 28.3% and the mandibular pattern in only 6.7% (Table 1).

**Table 1:** Demographic distribution of study participants

Variable	Chemical Peel, n (%)	Microneedling, n (%)	Overall, n (%)
Age (years)			
18–30	8 (13.3%)	10 (16.7%)	18 (15.0%)
31–40	22 (36.7%)	20 (33.3%)	42 (35.0%)
41–50	24 (40.0%)	22 (36.7%)	46 (38.3%)
>50	6 (10.0%)	8 (13.3%)	14 (11.7%)
Gender			
Female	52 (86.7%)	54 (90.0%)	106 (88.3%)
Male	8 (13.3%)	6 (10.0%)	14 (11.7%)
Pattern			
Centrofacial	38 (63.3%)	40 (66.7%)	78 (65.0%)
Malar	18 (30.0%)	16 (26.7%)	34 (28.3%)
Mandibular	4 (6.7%)	4 (6.7%)	8 (6.7%)
Total	60 (100%)	60 (100%)	120 (100%)

At baseline, the mean MASI score in the chemical peel group was 22.85 ± 4.35, which was substantially higher compared with the microneedling group (9.18 ± 4.10), and the between-group difference was highly significant (p < 0.001). By week 4, both groups demonstrated a significant reduction in MASI scores; chemical peel patients improved to 18.30 ± 3.90 (p < 0.001 within group), while microneedling patients reduced further to 6.12 ± 2.46 (p < 0.001 within group). At week 8, the trend of improvement continued, with MASI scores declining to 15.05 ± 3.82 in the chemical peel group and 5.40 ± 2.30 in the microneedling group, again significant within groups and between groups (p < 0.001). By week 12, the chemical peel group showed a mean MASI score of 12.92 ± 3.60, whereas the microneedling group achieved a lower mean of 5.09 ± 2.02 (Table 2).

**Table 2:** MASI score progression over the study period MASI: Modified Melasma Area and Severity Index.

Assessment Time	Chemical Peel (n = 60), Mean ± SD	Microneedling (n = 60), Mean ± SD	p-value (Within Groups)	p-value (Between Groups)
Baseline	22.85 ± 4.35	9.18 ± 4.10	–	<0.001
4 weeks	18.30 ± 3.90	6.12 ± 2.46	<0.001	<0.001
8 weeks	15.05 ± 3.82	5.40 ± 2.30	<0.001	<0.001
12 weeks	12.92 ± 3.60	5.09 ± 2.02	<0.001	<0.001

At 12 weeks, 30% (n = 18) of microneedling patients achieved ≥60% improvement compared with 16.7% (n = 10) in the peel group. Moderate improvement (40-59%) was also more frequent in the microneedling group (36.7% vs. 26.7%). Conversely, lower response rates were more commonly observed among patients treated with chemical peels, as 23.3% demonstrated less than 20% improvement compared with eight patients (13.3%) in the microneedling group, and 20 patients (33.3%) achieved only 20-39% improvement compared with 12 patients (20.0%) in the microneedling group (Table 3).

**Table 3:** Proportion of patients achieving specific improvement ranges

MASI Improvement, %	Microneedling, n (%)	Chemical Peel, n (%)
<20	8 (13.3%)	14 (23.3%)
20–39	12 (20.0%)	20 (33.3%)
40–59	22 (36.7%)	16 (26.7%)
≥60	18 (30.0%)	10 (16.7%)
Total	60 (100%)	60 (100%)

Adverse events were more frequent in the chemical peel group (35 patients, 58.3%) compared with the microneedling group (22 patients, 36.7%). Temporary redness was the most common side effect, affecting 16 patients (26.7%) in the chemical peel group and 10 patients (16.7%) in the microneedling group. Burning sensation occurred in 12 patients (20.0%) receiving chemical peels, whereas only five patients (8.3%) reported it in the microneedling group. Mild swelling was observed in four patients (6.7%) in the chemical peel group and three patients (5.0%) in the microneedling group. Itching was reported in four patients (6.7%) from the microneedling group and three patients (5.0%) from the chemical peel group (Table 4).

**Table 4:** Adverse effects reported during the study

Adverse Effect	Microneedling, n (%)	Chemical Peel, n (%)
Temporary redness	10 (16.7%)	16 (26.7%)
Burning sensation	5 (8.3%)	12 (20.0%)
Mild swelling	3 (5.0%)	4 (6.7%)
Itching	4 (6.7%)	3 (5.0%)
Any adverse event	22 (36.7%)	35 (58.3%)

Most participants in the study had darker skin phototypes, with Fitzpatrick type IV being the most common, observed in 26 patients (43.3%) in the microneedling group and 28 patients (46.7%) in the chemical peel group, accounting for a total of 54 patients (45.0%). Type V was the next most frequent, equally represented in both groups with 24 patients (40.0%) each, making up 48 patients (40.0%) overall. Type III was less common, seen in 10 patients (16.7%) undergoing microneedling and eight patients (13.3%) treated with chemical peels, totaling 18 patients (15.0%) (Table 5).

**Table 5:** Fitzpatrick skin phototype distribution

Skin Type	Microneedling, n (%)	Chemical Peel, n (%)	Total, n (%)
Type III	10 (16.7%)	8 (13.3%)	18 (15.0%)
Type IV	26 (43.3%)	28 (46.7%)	54 (45.0%)
Type V	24 (40.0%)	24 (40.0%)	48 (40.0%)
Total	60 (100%)	60 (100%)	120 (100%)

## Discussion

This study demonstrated that both microneedling with tranexamic acid and vitamin C and 15% TCA chemical peeling significantly improved melasma severity over 12 weeks. However, microneedling achieved greater overall pigment reduction, higher rates of patient-reported improvement, and fewer adverse effects, highlighting its superiority as a first-line procedural treatment for moderate to severe melasma. Nevertheless, the microneedling group demonstrated greater mean reductions in MASI scores consistently at all follow-ups, with the final average score compared with 13.16 ± 4.49 in the peel group. This was not only statistically significant but also clinically meaningful because of the increased dermal penetration and focus of action offered by microneedling-aided transdermal delivery. The effectiveness of microneedling in this study agrees with earlier studies that showed the superiority of tranexamic acid administered by using microneedling, with reductions in the MASI score being higher than with chemical peels or other topical treatments [22]. A proportion of 30% of microneedling patients had 60% improvement or more, which was higher than that of the chemical peel group (16.7%). These results are comparable to those of past studies in which patient and physician global assessment scores were higher in microneedling arms relative to chemical peels, especially in patients with darker skin phototypes.

The demographic pattern of our study, mainly of female subjects (88.3%) and Fitzpatrick skin types IV and V, can be supported by other studies done in the epidemiology of melasma and supports the applicability of these treatment modes to affected individuals due to their inclination toward postinflammatory hyperpigmentation [23]. The most frequent centrofacial pattern (64.2% in total) also concurs with the clinical patterns often mentioned in the previous literature. TCA peels, with notable changes in MASI scores (ΔMASI: −9.81 ± 4.93), had higher rates of transient erythema (40%) and burning (26.7%) than microneedling (26.7% and 16.7%, respectively). This observation aligns with other studies that showed that mild to moderate-depth TCA peels frequently yield transient irritation, though they remain an economical solution, particularly in resource-limited areas [24][25].

The property of microneedling to form homogeneous microchannels enables deeper delivery of tranexamic acid, which inhibits melanogenesis by slowing the production of plasmin and subsequent stimulation of tyrosinase in melanocytes. Vitamin C provides an antioxidant effect, guarding against the oxidative stimulation of melanocytes that induces melanin formation. These two mechanisms likely explain the better pigment reduction observed in our trial. Combination treatments used in other research have also been reported as very useful in managing refractory cases of melasma [26]. Notably, both therapies had a positive safety profile without any critical safety events, which is consistent with the safety rates reported in related studies [27]. Nevertheless, the increased relative response of higher-grade improvement in the microneedling group, as well as its lower irritation profile, suggests that it may provide a superior alternative as a first-line procedural treatment in those with moderate to severe melasma, especially in darker phototypes with an increased risk of pigmentary complications.

Limitations

This study has valuable findings, but it must be acknowledged that it has several limitations. First, the study was observational and not randomized; the absence of randomization and allocation concealment introduces a potential risk of selection bias, which makes selection bias more likely and makes it harder to establish whether a treatment modality and clinical outcomes are linked. Second, the follow-up period lasted only 12 weeks, which may not adequately capture the long-term efficacy and relapse rates of melasma, a condition known for its chronic and recurrent nature. Third, the study was carried out at a single tertiary care facility with a relatively uniform patient population, the majority of whom were females with Fitzpatrick skin types IV and V. As a result, the results may not be applicable to other populations or skin types.

## Conclusions

Both 15% TCA peeling and microneedling with tranexamic acid plus vitamin C effectively reduced the severity of moderate to severe melasma. However, microneedling achieved greater reductions in MASI scores, higher rates of clinically meaningful improvement, and fewer transient side effects compared with TCA peeling. These findings indicate that microneedling-assisted delivery of tranexamic acid and vitamin C offers a more effective, safer, and better-tolerated treatment option, particularly for individuals with darker skin phototypes or those at increased risk of postinflammatory hyperpigmentation. While TCA peeling remains a cost-effective and accessible method, microneedling may be preferred when aiming for superior pigment clearance and patient satisfaction.

## References

[1] Chauhan DA (2019). A comparative study to evaluate the efficacy of trichloroacetic acid versus gycolic acid chemical peels in treatment of melasma. J Med Sci Clin Res.

[2] Halder RM, Kindred C (2008). Faculty opinions recommendation of: successful treatment of moderate to severe melasma with triple-combination cream and glycolic acid peels: a pilot study. Faculty Opinions.

[3] Shams SA, Suman S, Hussain S, Viplav V (2023). A comparative study of clinical efficacy of 35% glycolic acid and 20% salicylic acid peels in melasma. J Med Sci Clin Res.

[4] Pooja P, Kaur T, Malhotra S (2021). Comparative evaluation of microneedling with meso solution versus microneedling alone in treatment of recalcitrant melasma: a split-face study. Int J Sci Res.

[5] Menon A, Eram H, Kamath PR, Goel S, Babu AM (2020). A split face comparative study of safety and efficacy of microneedling with tranexamic acid versus microneedling with Vitamin C in the treatment of melasma. Indian Dermatol Online J.

[6] Abdalla MA (2021). Melasma clinical features, diagnosis, epidemiology and etiology: an update review. Siriraj Med J.

[7] Artzi O, Horovitz T, Bar-Ilan E (2021). The pathogenesis of melasma and implications for treatment. J Cosmet Dermatol.

[8] Bertotti PP, Cé R (2024). Melasma: understanding its complexity and accurate therapeutic approaches. Rev Científica Sophia.

[9] Ali L, Al Niaimi F (2025). Pathogenesis of melasma explained. Int J Dermatol.

[10] Lazar M, De La Garza H, Vashi NA (2023). Exogenous ochronosis: characterizing a rare disorder in skin of color. J Clin Med.

[11] Maddaleno AS, Camargo J, Mitjans M, Vinardell MP (2021). Melanogenesis and melasma treatment. Cosmetics.

[12] Liu W, Chen Q, Xia Y (2023). New mechanistic insights of melasma. Clin Cosmet Investig Dermatol.

[13] Kania B, Lolis M, Goldberg D (2025). Melasma management: a comprehensive review of treatment strategies including BTX-A. J Cosmet Dermatol.

[14] Xu Y, Ma R, Juliandri J (2017). Efficacy of functional microarray of microneedles combined with topical tranexamic acid for melasma: a randomized, self-controlled, split-face study. Medicine (Baltimore).

[15] Hoque F, McGrath J, Shaude SE (2022). Melasma (Chloasma): pathogenesis and treatment. J Biotechnol Biomed.

[16] Cassiano DP, Espósito AC, da Silva CN (2022). Update on melasma - Part II: treatment. Dermatol Ther (Heidelb).

[17] Piętowska Z, Nowicka D, Szepietowski JC (2022). Understanding melasma - how can pharmacology and cosmetology procedures and prevention help to achieve optimal treatment results? A narrative review. Int J Environ Res Public Health.

[18] Ghasemiyeh P, Fazlinejad R, Kiafar MR, Rasekh S, Mokhtarzadegan M, Mohammadi-Samani S (2024). Different therapeutic approaches in melasma: advances and limitations. Front Pharmacol.

[19] Budamakuntla L, Loganathan E, Suresh DH (2013). A randomised, open-label, comparative study of tranexamic acid microinjections and tranexamic acid with microneedling in patients with melasma. J Cutan Aesthet Surg.

[20] Kimbrough-Green CK, Griffiths CE, Finkel LJ, Hamilton TA, Bulengo-Ransby SM, Ellis CN, Voorhees JJ (1994). Topical retinoic acid (tretinoin) for melasma in black patients. A vehicle-controlled clinical trial. Arch Dermatol.

[21] Mattei PL, Corey KC, Kimball AB (2014). Psoriasis Area Severity Index (PASI) and the Dermatology Life Quality Index (DLQI): the correlation between disease severity and psychological burden in patients treated with biological therapies. J Eur Acad Dermatol Venereol.

[22] Bostan E, Cakir A (2023). The dermoscopic characteristics of melasma in relation to different skin phototypes, distribution patterns and wood lamp findings: a cross-sectional study of 236 melasma lesions. Arch Dermatol Res.

[23] Galache TR, Sena MM, Tassinary JA, Pavani C (2024). Photobiomodulation for melasma treatment: integrative review and state of the art. Photodermatol Photoimmunol Photomed.

[24] Jo JY, Chae SJ, Ryu HJ (2024). Update on melasma treatments. Ann Dermatol.

[25] Gao TW, Gu H, He L (2021). Dermatologist Association Consensus on the diagnosis and treatment of melasma in China (2021 version). Int J Dermatol Venereol.

[26] Desai SR, Alexis AF, Elbuluk N, Grimes PE, Weiss J, Hamzavi IH, Taylor SC (2024). Best practices in the treatment of melasma with a focus on patients with skin of color. J Am Acad Dermatol.

[27] Winaya KK, Mahariski PA, Praharsini IG, Pramita IG (2023). Dermoscopic features of melasma: a descriptive study in Bali. Bali Med J.

